# Serum soluble DR5 predicts mortality risk in patients with HBV-related hepatocellular carcinoma

**DOI:** 10.3389/fonc.2022.1040812

**Published:** 2022-12-20

**Authors:** Jiaqi Liang, Ying Feng, Yao Liu, Ke Shi, Guiqin Zhou, Long Liu, Yaxin Liu, Kexin Qiao, Wen Liu, Xianbo Wang

**Affiliations:** ^1^ Department of Infectious Disease, Beijing Hospital of Traditional Chinese Medicine, Capital Medical University, Beijing, China; ^2^ Department of Integrative Medicine, Beijing Ditan Hospital, Capital Medical University, Beijing, China

**Keywords:** soluble DR5, hepatocellular carcinoma, prognosis, serum marker, mortality

## Abstract

**Introduction:**

Death receptor 5 (DR5) is significantly upregulated in various human tumor tissues; however, the relationship between serum levels of soluble DR5 (sDR5) and the mortality risk of hepatocellular carcinoma (HCC) is not understood. Our aim is to investigate the prognostic value of serum sDR5 in HCC patients.

**Methods:**

A total of 170 patients with HBV-HCC were recruited, with 82 and 88 patients as derivation and validation cohorts, respectively. sDR5 levels were analyzed using ELISA. The predictive factors for mortality were selected using LASSO regression analysis. Cox regression analysis was used to analyze the independent factors affecting mortality in 2 years. A nomogram based on the interquartile range of the sDR5 values predicted mortality rates.

**Results:**

Serum sDR5 level was identified as an independent risk factor for mortality in patients with HBV-HCC. The 2-year cumulative mortality rates of HBV-HCC were 10, 28.57, 38.10, and 95% across the sDR5 quartiles, respectively (p < 0.001). The sDR5 had an AUROC of 0.851 (95% CI: 0.755–0.920) in the derivation cohort. When the cut-off value was 30.06pg/mL, the AUROC of sDR5 was 0.778 (95% CI 0.677–0.860) in the validation cohort. The calibration curves fit well, and the decision curves showed that sDR5 had a high standardized net benefit. sDR5 predicted the prognosis of HBV-HCC patients most accurately. Further, serum sDR5 level was significantly positively associated with BCLC stage and the presence or absence of ascites.

**Conclusion:**

sDR5 showed high predictive accuracy in patients with HBV-HCC; thus, it is considered a new serological biomarker.

## 1 Introduction

Primary liver cancer (PLC) is the sixth most commonly diagnosed cancer, the third leading cause of cancer death worldwide in 2020 (1), and the second leading cause of cancer death in mainland China (2, 3). Hepatocellular carcinoma (HCC) is the major histotype of PLC, and hepatitis B-related HCC accounts for more than 84.4% of all hepatocellular carcinomas (4), imposing heavy economic and social burdens on the country. Current treatment methods for HCC include surgery, transcatheter arterial chemoembolization (TACE), radiofrequency ablation (RFA), chemotherapy, molecular targeted therapies, and immune checkpoint inhibitor therapies. In addition, some new drugs for anticancer therapies are under development (5). However, most patients with HCC are already at an advanced stage when diagnosed, with limited treatment methods and poor prognosis, and the 5-year survival rate is only 15% (6). Therefore, an accurate judgment of the prognosis of patients with HCC is helpful for doctors to choose active or conservative clinical treatment strategies.

Serum biomarkers are easily obtained, convenient, and repeatable. Among them, serum alpha-fetoprotein (AFP) is a recognized indicator in the diagnosis and prognosis of HCC and associated with worse tumor phenotype and invasion in HCC (7). Some staging proposals for HCC also include AFP values, such as the Cancer of the Liver Italian Program (8), GRETCH staging from France (9), and the Chinese University Prognostic Index (10). However, a recent retrospective analysis found that approximately 31% of patients with confirmed HCC do not have a significant increase in serum AFP levels, which greatly limits its role in HCC diagnosis and prognosis prediction (11). In addition to AFP, some serological markers, such as alpha-fetoprotein LP-3 and des-γ-carboxy-prothrombin, have been confirmed to be associated with vascular invasion of HCC, but their cut-off values for predicting HCC prognosis are controversial (12). Therefore, it is of great clinical significance to search for new serum biomarkers as an effective supplement to HCC prognostic staging models, such as the Barcelona Clinic Liver Cancer (BCLC) clinical algorithm, the Hong Kong Liver Cancer group, and the Japan Integrated Scoring system, which do not contain serological biomarkers.

Death receptor 5 (DR5; TNFRSF10B), a member of the tumor necrosis factor-receptor superfamily (TNFRSF), contains an intercellular death domain that can be triggered by receipt of tumor-necrosis-factor-related apoptosis-inducing ligands (TRAIL/TNFSF10/APO-2L) and transduces apoptotic signals. The main form of DR5 is a type I transmembrane protein, but its extracellular domain can be expressed in plasma as soluble monomers, resulting in soluble DR5 (sDR5) (13). The overexpression of DR5 in various human tumors has been confirmed (14), including colorectal cancer (15, 16), lung cancer (17), cervical cancer (18), and head and neck squamous cell carcinoma (19). In addition, a previous study indicated that the upregulation of sDR5 levels was significantly correlated with poor prognosis of patients with non-small cell lung cancer (20), making sDR5 a potential serum biomarker for prognosis. However, the relationship between serum sDR5 expression in patients with HCC and their prognosis has not been reported.

In this study, we first verified that serum sDR5 is an independent prognostic factor for 2-year overall survival in patients with hepatitis-B virus (HBV)-HCC, and its ability to predict the 2-year survival rate of patients was also confirmed. Patients were then grouped according to the quartile of sDR5 levels, and the relationship between sDR5 expression and prognosis in each group was analyzed hierarchically. In addition, the optimal cutoff value of sDR5 for determining the 2-year prognosis of patients with HCC was determined and evaluated in an independent validated cohort. Moreover, the relationship between serum sDR5 levels and the typical clinicopathological features of HCC patients was analyzed. In summary, we sought to provide information on the prognostic value of sDR5 as a novel biomarker for patients with HCC.

## 2 Patients and methods

### 2.1 Patients

**Figure 1:**
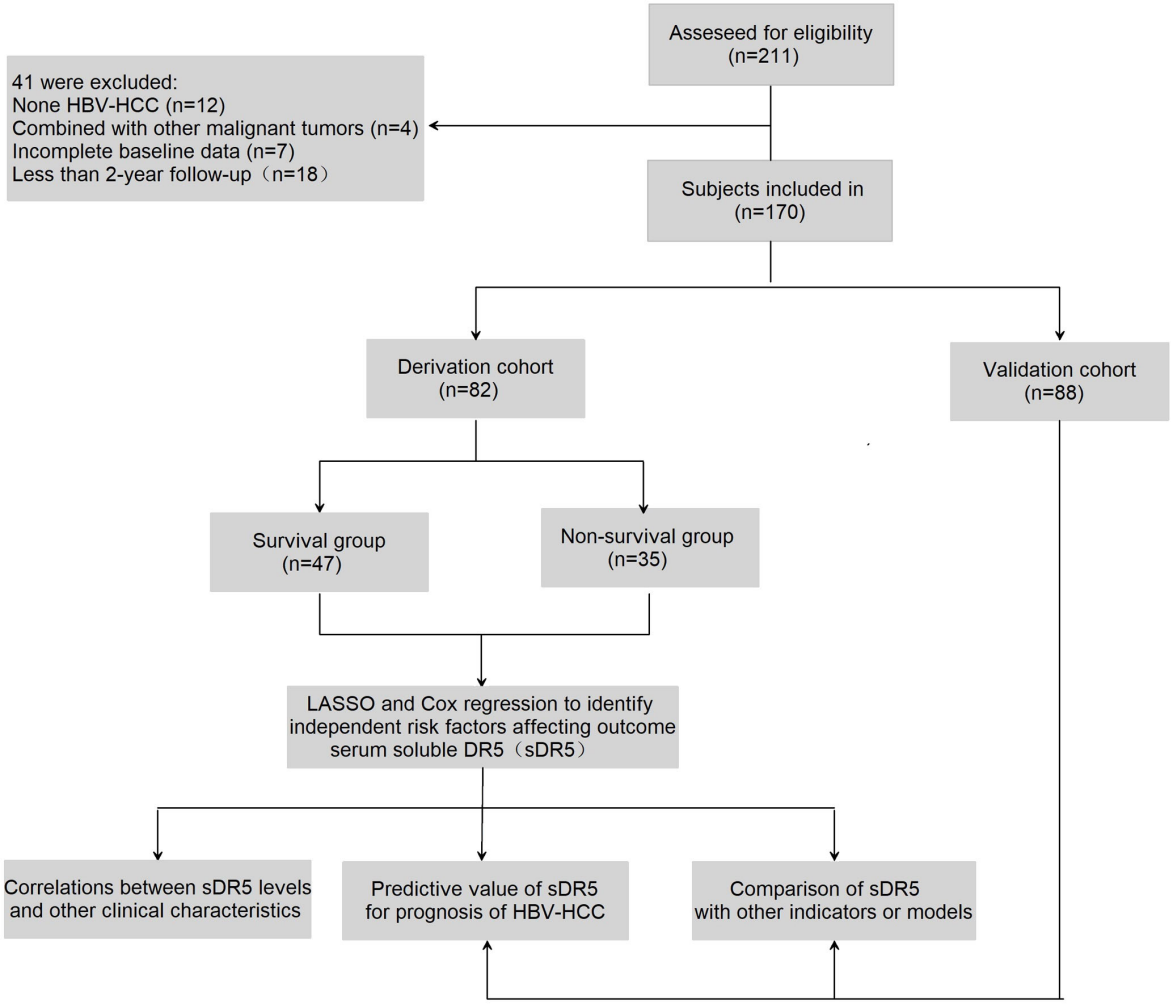
Flow chart of the clinical research. A total of 211 subjects were screened for eligibility, 170 patients were included in the research. Among them, 82 participants were selected in the derivation group and 88 in validation group.

A total of 211 patients with HCC were recruited from Beijing Ditan Hospital, Capital Medical University between March 2014 and July 2017; 170 patients were included in the study; among these, 82 patients were enrolled in the derivation cohort between March 2014 and November 2014, and 88 patients were enrolled in the validation cohort between January and July 2017(**Figure 1**). All participants were initially diagnosed with HBV-related HCC based on the recommendations of the American Association for the Study of Liver Diseases. The exclusion criteria are described in an earlier paper published by our research group (21). The cohort met the criteria for continuous oral nucleos(t)ide analogs (NAs) treatment for more than 6 months. All patients received standard treatment recommended by the guidelines during the follow-up period, such as RFA, TACE, and conservative treatments, according to the features of recurrent tumors and the patient’s liver function condition. This study was approved by the Institutional Research Ethics Committee of the Beijing Ditan Hospital, Capital Medical University (Beijing, China). All the patients provided signed informed consent.

### 2.2 Data collection

Clinical data included demographic status (age, sex, smoking, alcohol consumption, and family history of HCC), complications (ascites, hepatic encephalopathy), routine blood tests (white blood cell [WBC] count, neutrophil-to-lymphocyte ratio [NLR]), liver function (alanine aminotransferase [ALT], aspartate aminotransferase [AST], total bilirubin [TBIL], serum albumin [ALB], alkaline phosphatase [ALP], ɣ-glutamyl transpeptidase [GGT], Child–Pugh stage, MELD score), serum creatinine (Scr), international normalized ratio (INR), plasma prothrombin activity (PTA), virus replication (HBV DNA) and tumor-related indicators (AFP, tumor number, largest tumor diameter, lymphnode metastasis, portal vein tumor thrombosis, and BCLC staging). Data were obtained at the time of serum collection from patients with HCC.

### 2.3 Observation outcome

Patient survival was the endpoint of the study and was measured in days from the time of the initial diagnosis of HCC to death or the last follow-up date. The follow-up period was 2 years (range, 730 days). Patients who were not available for 2 years of follow-up were not included in the study.

### 2.4 Enzyme-linked immunosorbent assay

The level of serum sDR5 expression was measured using a commercial ELISA kit (lot no. JL20076, Jianglai Inc., Shanghai, China) according to the manufacturer’s instructions. The absorbance of the resultant solutions was measured at 450 nm using a microplate reader (Sigma Aldrich, St. Louis, MO, USA).

### 2.5 Statistical analysis

Statistical analyses were performed using SPSS (version 22.0; IBM, NY, USA), GraphPad 8.0 (GraphPad Software, CA, USA), software packages (glmnet, rms, survival, pheatmap) in R (version 4.1.3, R Foundation for Statistical Computing, Vienna, Austria), and MedCalc 17.9 (MedCalc Software, Belgium). The clinical and demographic characteristics in this study were summarized as median, range, and number. The enumeration data were statistically described by frequency and rate. The chi-square test was used to compare rates between the groups. Fisher’s exact test was used for those that did not meet the chi-squared test conditions. The measurement data showing a normal distribution are expressed as “mean ± standard deviation” (x ± s), while those showing a non-normal distribution are expressed as “median (interquartile range)” [M (p25–p75)]. The differences between the two groups were determined *via* a t-test for continuous variables with normal distribution and the Mann–Whitney U test for continuous variables that did not have a normal distribution. The Kaplan–Meier method was used to describe the survival curve. Statistical significance was set at p < 0.05, and all experiments were repeated at least thrice.

Least absolute shrinkage and selection operator (LASSO) regression was used to filter the variables by generating a penalty function to compress the variable coefficients in the regression model to prevent overfitting and solve the problem of serious collinearity. The optimum parameter (lambda) selection in the LASSO model was performed by tenfold cross-validation using the minimum criteria. The partial likelihood deviance (binomial deviance) curve is presented versus log (lambda). Dotted vertical lines are shown at the optimum values by performing lambda.min and lambda.1se. Multivariate Cox regression was used to identify independent risk factors affecting outcomes. The matrix heatmapshowed the expression of serum sDR5 in the different patient samples. A nomogram was established based on DR5 expression, which was used to predict mortality at 1, 2, and 3 years. Harrell’s concordance index was used to measure how well a variable or model predicted time to a censored event. The calibration curve reflects the relationship between the prediction rate and the actual occurrence rate. The decision curve was used to evaluate the clinical application of the model, which displays estimates of the standardized net benefit by the probability threshold used to categorize observations as “high-risk”. MedCalc software was used to create and compare the models.

## 3 Results

### 3.1 Baseline characteristics of patients

Overall, 170 patients diagnosed with HBV-related HCC were included in the study, of whom 82 were included in the derivation cohort. The derivation group comprised 74 men and 8 women aged 48.75–60 years, while the validation group comprised 76 men and 12 women aged 49–62.75 years. The clinical characteristics of the derivation and validation cohorts are summarized in **Table 1**. Except for age and ALP, there were almost no significant differences between the two cohorts in terms of sex, smoking, alcohol consumption, family history of HCC, complications, WBC, Child–Pugh stage, MELD score, serum creatinine, coagulation function, virus replication, or tumor-related indicators (**Table 1**).

**Table 1:** Clinical characteristics of patients with HBV-related hepatocellular carcinoma.

Variables	All (n=170)	Derivation cohort (n=82)	Validation cohort(n=88)	t/Z/*χ*2	*p* value
Age,year	56 (49-61.25)	54.5 (48.75-60)	57 (49-62.75)	0.267	0.015 ^b)^
Sex Male	150 (88.24)	74 (90.24)	76 (86.36)	0.616	0.433 ^c)^
Smoking	91 (53.53)	44 (53.66)	47(53.41)	0.001	0.974 ^c)^
Alcohol	88(51.76)	42 (51.22)	46 (52.27)	0.019	0.891 ^c)^
Family history	34 (20.00)	20 (24.39)	14 (15.91)	1.908	0.167 ^c)^
Acites	81 (47.65)	34(41.46)	47(53.41)	2.428	0.119 ^c)^
Ecephalopathy	7 (4.12)	2 (2.44)	5 (5.68)	0.458	0.498 ^c)^
WBC,×10^9^/L	4.49 (3.16-5.85)	4.67 (3.44-5.72)	4.29 (3.15-6.22)	-0.012	0.990 ^b)^
NLR	2.81 (1.83-4.73)	2.85 (1.87-4.73)	2.77 (1.79-4.75)	-0.089	0.929 ^b)^
ALT,u/L	32.9 (20.65-46.05)	32.65 (21.33-43.83)	33.7 (20.05-50.03)	-0.033	0.974 ^b)^
AST,u/L	37.3 (28.2-70.3)	38.15 (30.75-62.93)	36.3 (24.85-76.38)	-0.825	0.409 ^b)^
TBIL,μmol/L	21.05 (13.45-38.18)	21.1 (15.15-38.18)	21 (13.13-38.23)	-0.137	0.891 ^b)^
ALB,g/L	36.42±6.6	35.84±7.26	36.96±5.9	0.187	0.268 ^a)^
ALP,u/L	90.95 (67.28-136.45)	83.45 (61.78-129.85)	95.9 (74.98-151.13)	-2.361	0.018 ^b)^
GGT,u/L	56.6 (26.73-112.7)	56.95 (28.6-102.5)	56.6 (25.58-133.65)	-0.125	0.901 ^b)^
Cr, μmol/L	64.35 (56-77.03)	63.2 (55.83-77)	64.95 (56.05-78.53)	-0.405	0.685 ^b)^
PTA,%	79.7 (67-91)	79.7 (65.9-90.3)	79.5 (67.25-91.75)	-0.412	0.680 ^b)^
INR	1.17 (1.07-1.29)	1.13 (1.06-1.29)	1.18 (1.08-1.33)	-1.28	0.201 ^b)^
AFP≥400, ng/mL	39 (22.94)	14 (17.07)	25 (28.41)	2.085	0.079 ^c)^
HBVDNA≥500, IU/mL	52 (30.59)	25 (30.49)	27 (30.68)	0.01	0.978 ^c)^
Tumor number≥3	54 (31.76)	24 (29.27)	30 (34.09)	0.455	0.500 ^c)^
Largest tumor diameter≥5cm	35 (20.59)	15 (18.29)	20 (22.73)	0.511	0.475 ^c)^
Lymphnode metastasis	41 (24.12)	20(24.39)	21 (23.86)	0.006	0.936 ^c)^
Portal Vein Tumor Thrombosis	59(34.71)	23(28.05)	36 (40.91)	3.098	0.078 ^c)^
sDR5,pg/mL	33.89 (20.51-70.67)	43.94 (23.00-74.02)	28.32 (19.15-61.06)	-1.806	0.071 ^b)^
BCLC stage, 0-A	74 (43.53)	40 (48.78)	34 (38.64)	1.777	0.183 ^c)^
BCLC stage, B	26 (15.29)	13 (15.85)	13 (14.77)	0.038	0.845 ^c)^
BCLC stage, C-D	70 (41.18)	29 (35.37)	41 (46.59)	2.208	0.137 ^c)^
Child-Pugh stage, 1	98 (57.65)	46 (56.1)	52 (59.09)	0.156	0.693 ^c)^
Child-Pugh stage, 2	46 (27.06)	25 (30.49)	21 (23.86)	0.944	0.331 ^c)^
Child-Pugh stage, 3	26 (15.29)	11 (13.41)	15 (17.05)	0.432	0.511 ^c)^
MELD Score	6.16 (3.24-9.73)	6.01 (3.47-9.64)	6.28 (3.13-10.26)	-0.317	0.752 ^b)^

Data are presented as n (%), mean value (standard deviation), or median (interquartile range). p values comparing groups are from t test ^a)^ , Mann-Whitney U test ^b)^ , or χ² test ^c)^. WBC, white blood cell count. NLR, neutrophil-to-lymphocyte ratio. ALT, alanine aminotransferase. AST, aspartate aminotransferase. TBIL, total bilirubin. ALB, albumin. ALP, alkaline phosphatase. GGT, gamma-glutamyl transferase; Cr, creatinine. PTA, prothrombin activity. INR, international normalized ratio. AFP, α-fetoprotein. HBV, hepatitis B virus. BCLC, Barcelona Clinic for Liver Cancer. sDR5, soluble death receptor 5.

In the derivation cohort, the baseline characteristics of patients in the survival and non-survival groups were compared, and the results showed that in addition to age, sex, smoking, alcohol consumption, family history, encephalopathy, ALT level, serum Cr level, lymph node metastasis, and number of patients in BCLC stage B, most of the baseline characteristics of patients were different (**Table 2**).

**Table 2:** Clinical characteristics of patients with HBV-related hepatocellular carcinoma.

Variables	Survival (n=47)	Non-survival (n=35)	t/Z/*χ*2	*p* value
Age,year	56 (50-62)	54 (45-59)	-1.62	0.105 ^b)^
Sex Male	43 (91.49)	31 (88.57)	0.004	0.949 ^c)^
Smoking	28 (59.57)	16 (45.71)	1.55	0.213 ^c)^
Alcohol	24 (51.06)	18 (51.43)	0.001	0.974 ^c)^
Family history	10(21.28)	10 (28.57)	0.579	0.447 ^c)^
Acites	9 (19.15)	25 (71.43)	22.59	<0.001 ^c)^
Ecephalopathy	0 (0)	2 (5.71)	2.719	0.179 ^c)^
WBC,×10^9^/L	4.49 (3.05-5.27)	5.59 (3.49-6.47)	-2.348	0.019 ^b)^
NLR	2.09 (1.64-3.56)	4.29 (2.69-6.23)	-4.191	<0.001 ^b)^
ALT,u/L	32.8 (20.5-41.9)	28.7 (21.4-56.2)	-0.581	0.561 ^b)^
AST,u/L	33.4 (29.1-41.6)	62 (34.2-131.7)	-4.102	<0.001 ^b)^
TBIL,μmol/L	17.8 (11.6-25.6)	29.3 (18.4-78.5)	-3.239	0.001 ^b)^
ALB,g/L	38.63±7	32.09±5.85	0.246	<0.001 ^a)^
ALP,u/L	66.9 (58.9-87.7)	128.8 (79.9-169.1)	-4.284	<0.001 ^b)^
GGT,u/L	37.8 (21.6-65.3)	102.4 (50.3-224.1)	-4.467	<0.001 ^b)^
Cr, μmol/L	63.3 (55.3-77)	63.1 (56-77)	-0.028	0.978 ^b)^
PTA,%	83 (73.3-92)	71 (60.8-83.8)	-2.542	0.011 ^b)^
INR	1.11 (1.04-1.21)	1.2 (1.09-1.38)	-2.49	0.013 ^b)^
AFP≥400, ng/mL	2 (4.26)	12 (34.29)	10.745	0.001 ^c)^
HBVDNA≥500, IU/mL	9 (19.15)	16 (45.71)	6.68	0.010 ^c)^
Tumor number≥3	5 (10.64)	19 (54.29)	18.461	<0.001 ^c)^
Largest tumor diameter≥5cm	3 (6.38)	12 (34.29)	8.666	0.003 ^c)^
Lymphnode metastasis	9 (19.15)	11 (31.43)	1.64	0.200 ^c)^
Portal Vein Tumor Thrombosis	6 (12.77)	17 (48.57)	12.744	<0.001 ^c)^
sDR5,pg/mL	29.66 (12.98-47.73)	78.87 (45.73-440.23)	-5.414	<0.001 ^c)^
BCLC stage, 0-A	34 (72.34)	6 (17.14)	24.463	<0.001 ^c)^
BCLC stage, B	6 (12.77)	7 (20)	0.787	0.375 ^c)^
BCLC stage, C-D	7 (14.89)	22 (62.86)	20.19	<0.001 ^c)^
Child-Pugh stage, 1	38 (80.85)	8 (22.86)	27.396	<0.001 ^c)^
Child-Pugh stage, 2	8 (17.02)	17 (48.57)	9.423	0.002 ^c)^
Child-Pugh stage, 3	1 (2.13)	10 (28.57)	9.908	0.002 ^c)^
MELD Score	5.5 (2.32-7.4)	9.11 (4.28-13.81)	-2.902	0.004 ^b)^

Data are presented as n (%), mean value (standard deviation), or median (interquartile range). p values comparing groups are from t test ^a)^ , Mann-Whitney U test ^b)^ , or χ² test ^c)^. WBC, white blood cell count. NLR, neutrophil-to-lymphocyte ratio. ALT, alanine aminotransferase. AST, aspartate aminotransferase. TBIL, total bilirubin. ALB, albumin. ALP, alkaline phosphatase. GGT, gamma-glutamyl transferase. Cr, creatinine. PTA, prothrombin activity. INR, international normalized ratio. AFP, α-fetoprotein. HBV, hepatitis B virus. BCLC, Barcelona Clinic for Liver Cancer. sDR5, soluble death receptor 5.

### 3.2 Predictive factors of mortality

**Figure 2:**
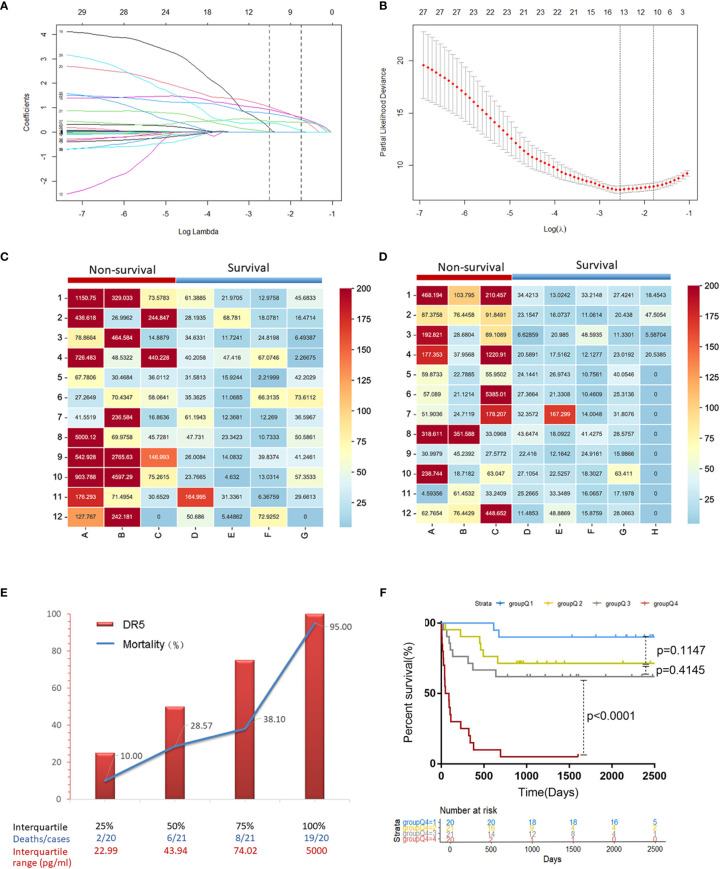
The predictive factor sDR5 was selected using LASSO regression analysis, and the correlation between serum sDR5 levels and mortality of patients with HBV-related HCC. **(A)** The LASSO coefficient profiles of the clinical features. **(B)** Optimum parameter (lambda) selection in the LASSO model performed with ten-fold cross-validation. The Matrix heatmap shows expression of sDR5 in patients with different prognosis in derivation cohort **(C)** and validation cohort **(D)**. **(E)** Comparison of mortality in HBV-HCC patients between four different concentration gradients of serum sDR5 based on the interquartile range. **(F)** Survival curves of the mortality in four groups based on the interquartile range of DR5 values using the Kaplan–Meier method.

Twenty-eight variables were included in the LASSO regression analysis: demographic status (age, sex, smoking, alcohol consumption, and family history), complications (ascites, encephalopathy), routine blood tests (WBC, NLR), liver function (ALT, AST, TBIL, ALB, ALP, GGT, Child-Pugh, MELD score), kidney function (serum creatinine), coagulation index (INR, PTA), viral replication (HBV DNA), tumor-related indicators (AFP, tumor number, largest tumor diameter, lymph node metastasis, portal vein tumor thrombosis, and BCLC staging), and serum sDR5 level. Two vertical lines were drawn at lambda.min and lambda.1se to screen for 10 predictors with non-zero coefficients, including sDR5 level, WBC, TBIL, ALP, GGT, ascites, AFP level, tumor number, Child-Pugh stage, and BCLC stage (**Figures 2A, B**).

These factors were included in the multivariate Cox regression analysis. Finally, sDR5 (HR = 1.001, 95% CI: 1–1.001, *p* = 0.030), WBC count (HR = 1.220, 95% CI: 1.001–1.488, *p* = 0.049), ascites (HR = 3.731, 95% CI: 1.190–11.691, *p* = 0.024), AFP ≥ 400ng/ml (HR = 4.458, 95% CI: 1.597–12.441, *p* = 0.004), and tumor number ≥ 3 (HR =3.1, 95% CI: 1.026–9.365, *p* = 0.045) were selected as independent prognostic factors for mortality after a follow-up of 2 years (**Table 3**).

**Table 3:** Multivariate Cox regression analyses for overall survival of patients with HBV-related hepatocellular carcinoma in the derivation cohort.

Variables	B	Wald	*P value*	OR (95%CI)
sDR5, pg/mL	0.001	4.719	0.030	1.001 (1-1.001)
WBC,×10^9^/L	0.199	3.882	0.049	1.22 (1.001-1.488)
TBIL,μmol/L	0.006	3.598	0.058	1.006 (1-1.012)
ALP,u/L	0.002	0.920	0.337	1.002 (0.997-1.008)
GGT,u/L	0.001	0.281	0.596	1.001 (0.998-1.004)
Acites	1.317	5.103	0.024	3.731 (1.19-11.691)
AFP ≥ 400, ng/mL	1.495	8.147	0.004	4.458 (1.597-12.441)
Tumor number ≥3	1.131	4.022	0.045	3.1 (1.026-9.365)
BCLC stage, 0-A		Ref	Ref	(-)
BCLC stage, B	-0.407	0.245	0.621	0.665 (0.132-3.344)
BCLC stage, C-D	0.406	0.395	0.530	1.501 (0.423-5.332)
Child-Pugh stage, 1		Ref	Ref	(-)
Child-Pugh stage, 2	0.453	0.546	0.460	1.573 (0.473-5.233)
Child-Pugh stage, 3	0.981	1.778	0.182	2.668 (0.631-11.285)

sDR5, soluble death receptor 5. TBIL, total bilirubin. ALP, alkaline phosphatase. GGT, gamma-glutamyl transferase. AFP, α-fetoprotein. BCLC, Barcelona Clinic for Liver Cancer.

**Figure 3:**
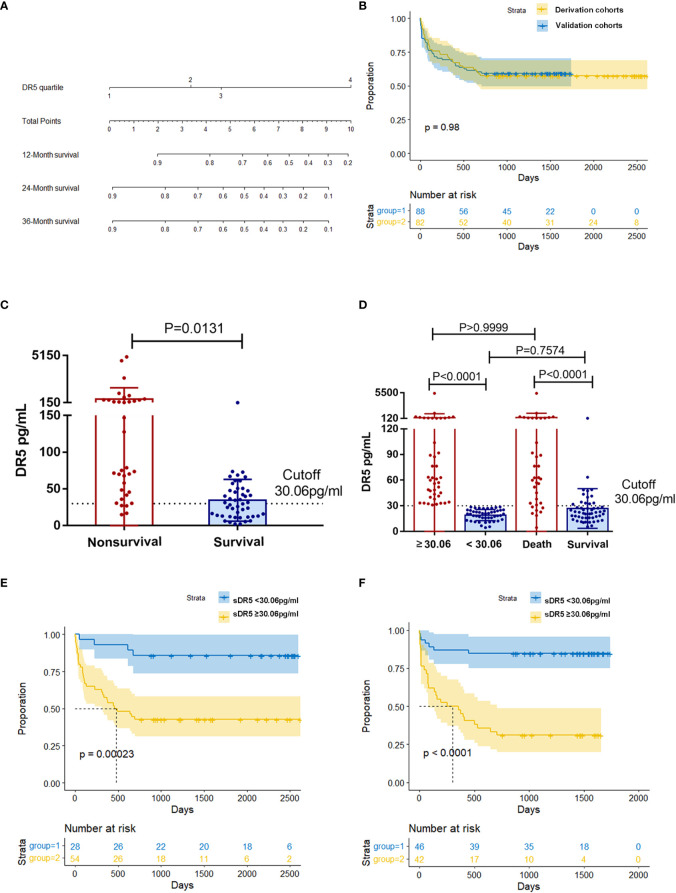
Relationship between different expression levels of sDR5 and 2-year prognosis in patients with HCC. **(A)** Nomogram of sDR5 predicting 1-, 2-, and 3-year overall survivals of patients with HCC. **(B)** Kaplan–Meier estimates of overall survival in the derivation and validation samples. Comparison of sDR5 expression levels between survival and non-survival groups within 2 years in derivation **(C)** and validation cohort **(D)**. Difference in sDR5 expression between high/low expression group and survival/non-survival group were compared in the validation cohort **(D)**. Kaplan–Meier estimates of overall survival between high/low expression group in the derivation **(E)** and validation **(F)** cohort when using cutoff value 30.06pg/mL.

In two independent cohorts of the derivation and validation groups, the matrix heatmap showed that patients who died within 2 years had higher serum levels of sDR5 (**Figures 2C, D**). We then divided the sDR5 levels of HCC patients into four concentration gradients based on the interquartile range and compared the 2-year mortalities of the different sDR5 concentration groups in derivation group. As the serum sDR5 concentration increased, the 2-year mortality of patients with HBV-HCC increased significantly from 10% to 95% (**Figure 2E**). Survival curves were calculated to compare the mortality in the four groups based on the interquartile range of sDR5 values using the Kaplan–Meier method, and the results showed a significant discriminatory ability for death risk in the patients (*p* < 0.0001, **Figure 2F**). The nomogram was also established based on the interquartile range of DR5 values, which were used to predict mortality rates at 1, 2, and 3 years (**Figure 3A**).

### 3.3 Predictive value of serum DR5 for prognosis of HBV-HCC

There was no significant difference in the sDR5 expression levels (*p* = 0.071, **Table 1**) or overall survival (*p* = 0.98, **Figure 3B**) between the derivation and validation groups. We further explored the relationship between serum sDR5 expression and survival time. In the derivation cohort, HCC patients with survival times of more than 2 years after diagnosis had significantly lower sDR5 levels than patients who died within 2 years (*p* = 0.0131, **Figure 3C**). Using the 2-year incidence of mortality as an endpoint, the grading of serum sDR5 high/low levels was determined using ROC curve analyses. When the optimal cut-off value was 30.06 pg/mL, death occurred in 14.29% (4/28) of patients in the sDR5 < 30.06 pg/mL strata and 57.41% (31/54) of patients in the sDR5 ≥ 30.06 pg/mL strata (*p* = 0.00023, **Figure 3E**). **Figure 3D** shows that in the validation cohort, HCC patients with survival times of more than 2 years after diagnosis also had significantly lower sDR5 levels than patients who died within 2 years (*p* < 0.0001). When the patients were divided into high and low-expression groups using the cutoff value of 30.06 pg/mL, there was no significant difference in sDR5 expression between the low-expression group and the survival group (*p* = 0.7574). The same trend was found in the comparison between the high-expression group and the death group (*p* > 0.9999). When the optimal cut-off value was 30.06 pg/mL, deaths occurred in 15.22% (7/46) of patients with sDR5 < 30.06 pg/mL, and 69.05% (29/42) of patients with sDR5 ≥ 30.06 pg/mL (*p* < 0.0001, **Figure 3F**).

**Figure 4:**
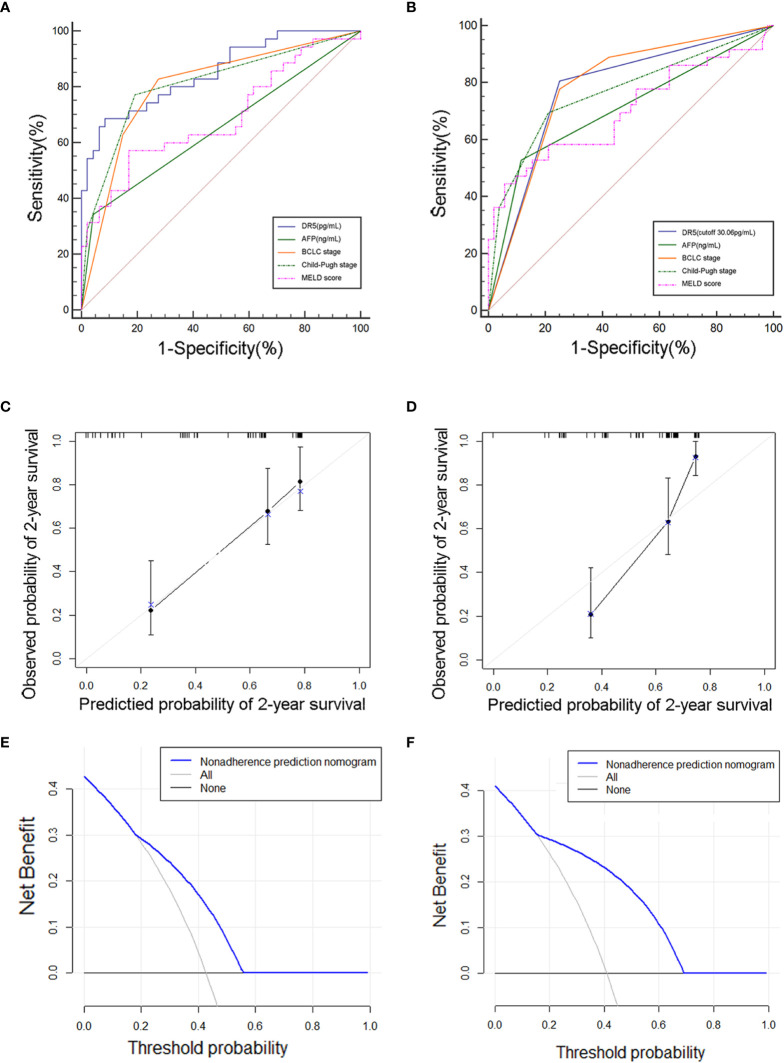
Evaluation of the prediction effect of cutoff value of sDR5 in the derivation **(A, C, E)** and validation **(B, D, F)** cohorts. **(A, B)** Receiver operating characteristic curve for the sDR5 and other model or indicators. **(C, D)** Calibration plot, **(E, F)** Decision curve.

Furthermore, the results showed an optimal cut-off value of 30.06 pg/mL, and the sensitivities and specificities of sDR5 for determining HBV-HCC mortality were 88.6% and 51.1% in the derivation cohort and 80.6% and 75.0% in the validation cohort, respectively. The concordance index (c-index) was 0.851 (95% CI: 0.755–0.920) for predicting mortality in the derivation cohort and 0.778 (95% CI: 0.677–0.860) in the validation cohort (**Figures 4A, B**).

The calibration curves showed that the predicted rates agreed with the actual results observed in the derivation (**Figure 4C**) and validation cohorts (**Figure 4D**). The decision curve showed that sDR5 had superior standardized net benefit on patient outcome (**Figures 4E, F**).

### 3.4 Comparison of serum sDR5 with other indicators or models

Using ROC analysis, the prognostic value of sDR5 for the incidence of mortality was compared with that of other indicators or models. sDR5 had the highest AUROC (0.851, 95% CI: 0.755–0.920) and higher sensitivity (0.886, 95% CI: 0.733–0.968) than AFP, BCLC stage, Child-Pugh stage, and MELD score in the derivation cohort (**Table 4**). In the validation cohort, the AUROC of sDR5 was 0.778 (95% CI: 0.677–0.860), the sensitivity was 0.806 (95% CI: 0.640–0.918), and the specificity was 0.750 (95% CI: 0.611–0.860, **Table 5**).

**Table 4:** Predictive value of the DR5, AFP, BCLC stage, Child-pugh stage and MELD score in derivation cohort.

Variables	AUC	Sensitivity(%)	Specificity(%)	Area difference with DR5	*P* value
	(95% CI)	(95% CI)	(95% CI)	(95% CI)	(95% CI)
sDR5,pg/mL	0.851	88.6	51.1	Ref	Ref
	0.755 - 0.920	73.3 - 96.8	36.1 - 65.9	Ref	Ref
AFP≥400, ng/mL	0.65	34.29	95.74	0.201	0.0001
	0.537 - 0.752	19.1 - 52.2	85.5 - 99.5	0.101 - 0.301	
BCLC stage	0.801	82.86	72.34	0.0498	0.3411
	0.698 - 0.881	66.4 - 93.4	57.4 - 84.4	-0.0528 - 0.152	
Child-Pugh stage	0.809	77.14	80.85	0.0419	0.4878
	0.707 - 0.888	59.9 - 89.6	66.7 - 90.9	-0.0765 - 0.160	
MELD	0.688	71.43	42.55	0.163	0.0161
	0.576 - 0.786	53.7 - 85.4	28.3 - 57.8	0.0302 - 0.296	

AUC, area under curve. sDR5, soluble death receptor 5. AFP, α-fetoprotein. BCLC, Barcelona Clinic for Liver Cancer.

**Table 5:** Predictive value of the DR5, AFP, BCLC stage, Child-pugh stage and MELD score in validation cohort.

Variables	AUC	Sensitivity (%)	Specificity (%)	Area difference with DR5	*P* value
	(95% CI)	(95% CI)	(95% CI)	(95% CI)	(95% CI)
sDR5≥30.06, pg/mL	0.778	80.6	75	Ref	Ref
	0.677 - 0.860	64.0-91.8	61.1-86.0	Ref	Ref
AFP≥400, ng/mL	0.706	52.78	88.46	0.0716	0.3352
	0.600 - 0.799	35.5-69.6	76.6-95.6	-0.0740~0.217	
BCLC stage	0.786	77.78	75	0.00855	0.9026
	0.686 - 0.867	60.8-89.9	61.1-86.0	-0.128~0.145	
Child-Pugh stage	0.766	69.44	78.85	0.0115	0.8634
	0.664 - 0.850	51.9-83.7	65.3-88.9	-0.119~0.142	
MELD	0.700	72.22	50	0.0780	0.2811
	0.593 - 0.793	54.8-85.8	35.8-64.2	-0.0638~0.220	

AUC, area under curve. sDR5, soluble death receptor 5. AFP, α-fetoprotein. BCLC, Barcelona Clinic for Liver Cancer.

### 3.5 Correlations between serum sDR5 levels and other clinical characteristics in HCC patients

**Figure 5:**
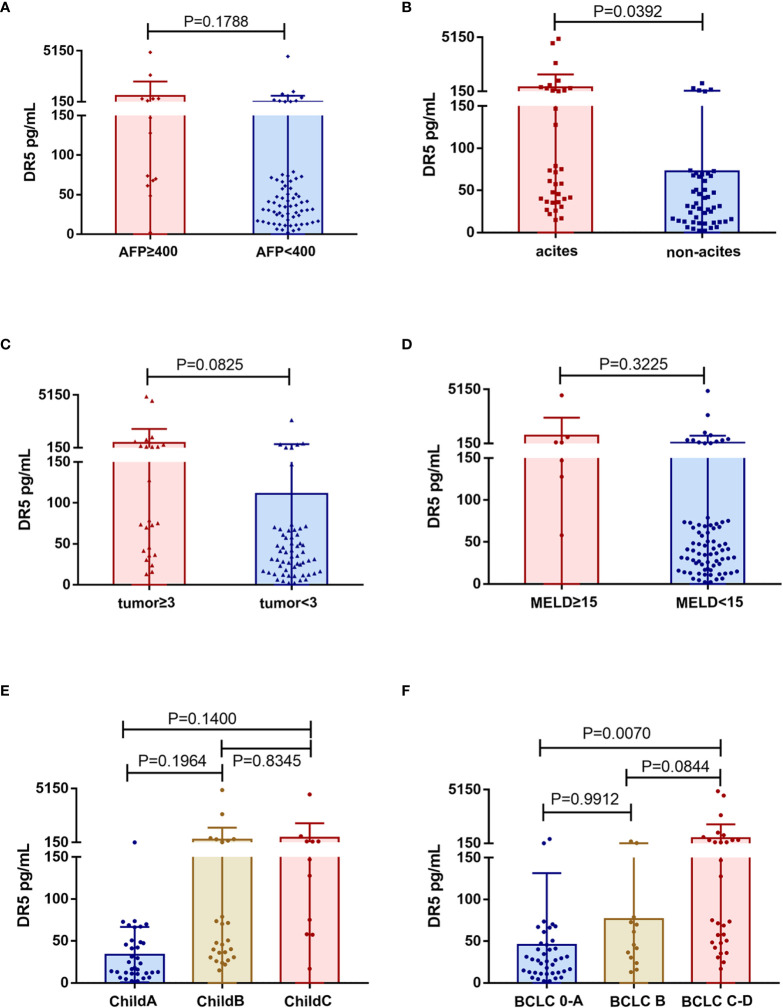
Association of serum levels of sDR5 with clinicopathological characteristics from HCC patients. **(A)** Serum level of sDR5 with AFP level **(A)**, ascites **(B)**, tumor number **(C)**, MELD score **(D)**, Child-Pugh stage **(E)**, and BCLC stage **(F)**.

Our data showed no association between sDR5 expression and AFP (*p =* 0.1788, **Figure 5A**), tumor number (*p =* 0.0825, **Figure 5C**), MELD score (*p =* 0.3225, **Figure 5D**), or Child-Pugh stage (*p >* 0.05, **Figure 5E**). However, serum sDR5 levels were significantly associated with ascites (*p =* 0.0392; **Figure 5B**) and BCLC stages 0-A and C (*p =* 0.0070; **Figure 5F**).

## 4 Discussion

Although researchers and clinicians have been working hard to improve the screening rate and treatment of HCC, the incidence rate and mortality of HCC have not significantly decreased, suggesting that HCC is still a refractory disease (3, 4). The evaluation of the prognosis of patients with HCC determines their follow-up frequency and treatment methods. Therefore, the accuracy of the prognostic evaluation system is important for HCC treatment.

Our study found that the serum sDR5 level, white blood cell count, presence of ascites, AFP level, and tumor number ≥ 3 were independent prognostic factors for HBV-HCC patients. An increase in WBC count is common in patients with HCC, often accompanied by an increase in granulocytes and a decrease in lymphocytes, suggesting immune suppression and gradual exhaustion of lymphocytes caused by repeated bacterial infections. The sustained local or systemic inflammatory response and the release of inflammatory factors mediated by pro-inflammatory macrophages and granulocytes can also promote the progression of HCC and cause death. The presence of ascites suggests that patients are in the decompensation stage of cirrhosis, and these patients are more likely to die of cirrhosis-related complications such as gastroesophageal variceal bleeding, hepatic encephalopathy, and spontaneous peritonitis. A number of intrahepatic tumors exceeding three suggests that the patient may have intrahepatic metastasis, and the effect of surgery and interventional therapy is poor. When the patients with HCC were divided into four groups according to the quartile of sDR5 expression, the Q4 group was associated with the highest risk of mortality, which was statistically significantly higher than in the Q3 group (HR = 6.291, 95% CI: 2.749–14.400, *p* < 0.0001). Another interesting finding is that although the expression level of sDR5 is closely related to BCLC stage and the 2-year prognosis of patients, there was no correlation between AFP and sDR5 expression. This suggests that DR5 can not only be used as an independent indicator for evaluating the prognosis of HCC but also as an effective supplement for judging the prognosis of AFP-negative HCC patients (accounting for 30% in total). In future research, we will continue to study the predictive value of sDR5, AFP, and BCLC staging and their combined application on the progression of HCC.

To date, five death receptors of the TNFRSF have been identified. Among these, only DR4 (TNFRSF10A) and DR5 (TNFRSF10B) have intracellular death domains. When combined with TNF-related apoptosis-inducing ligand (TRAIL), they can transmit apoptotic signals to the cell interior and initiate apoptosis (22). The expression of DR5 in tumors is higher than that in normal tissues, which may be related to the antitumor immune response of the body. Its extracellular domain is expressed in the form of soluble monomers and can be detected in blood (13) (23). It is generally believed that the increase in soluble DR5 in the patient’s serum is related to the expansion of tumor cells (20), but some also originates from neutrophils (24). In this study, we found that the expression of sDR5 was significantly correlated with BCLC stage (*p* = 0.0070) and the presence of ascites *(p* = 0.0392), with a trend toward correlation with the number of tumors (*p* = 0.0825), indicating that serum sDR5 expression may be closely related to tumor tissue. It should be noted that in addition to portal hypertension and hypoalbuminemia, peritoneal infection and intraperitoneal implantation of tumor are also common causes of ascites formation. Further analyses can be conducted to determine whether the increase in sDR5 is also related to bacterial infection.

In the derivation group, the AUROC of sDR5 was 0.851, suggesting that its ability to predict mortality was better than that of the BCLC stage (AUROC 0.801), AFP level (AUROC 0.650), Child-Pugh stage (AUROC 0.809), and MELD scores (AUROC 0.688). Based on the Yoden index, we determined 30.06pg/ml as the critical value for high and low levels of serum sDR5. Applying this cutoff value, in the validation group, the AUROC of sDR5 was lower than that of the BCLC score (0.778 vs. 0.786), but still superior to that of AFP (AUROC 0.706) and had the highest sensitivity (80.6%). Therefore, sDR5 may serve as a potential serological prognostic indicator.

This study had some limitations. First, the number of participants was small (82 and 88 in the derivation and validation cohorts, respectively). Second, as the age and ALP levels of patients in the derivation and validation cohorts were statistically different, the baseline was not completely balanced. In addition, because the patients in the validation group had a serious loss of follow-up rate after two years, the prognosis of the two groups of patients after two years was not obtained. Moreover, only the baseline data of the patients at the time of diagnosis were available, and there was no dynamic test to observe whether there was a change in sDR5 levels after treatment.

## 5 Conclusions

This is the first finding that the serum sDR5 is an independent prognostic factor for 2-year overall survival in patients with HBV-HCC. The sDR5 was sought to be provide as a novel biomarker for patients with HBV-HCC. We hope to carry out prospective, large-sample, multicenter studies in the future to further verify the advantages of sDR5 as a prognostic marker in HBV-HCC.

## Data availability statement

The raw data supporting the conclusions of this article will be made available by the authors, without undue reservation.

## Ethics statement

The studies involving human participants were reviewed and approved by Institutional Research Ethics Committee of the Beijing Ditan Hospital, Capital Medical University (Beijing, China). The patients/participants provided their written informed consent to participate in this study.

## Author contributions

XW and WL designed and supervised the entire study. JL and YF analyzed the clinical data and performed the experiments. YF wrote the manuscript. YL, LL and KS provided support for the analysis of clinical data. JL, YXL, KQ and GZ collected the clinical data. XW and YF obtained funding. All authors agree to be responsible for all aspects of their work, ensuring completeness and accuracy. All authors contributed to the article and approved the submitted version.
